# Whole-cell response of coronavirus-infected BMDCs through proteomic and transcriptomic analyses

**DOI:** 10.3389/fimmu.2025.1513952

**Published:** 2025-06-06

**Authors:** Yanxi Ji, Yuzhen Zhang, Yongjia Tong, Liu Cao, Lanyi Zeng, Yiran Xu, Xiao Guo, Tiefeng Xu, Zhidong Tang, Zhen Zhang, Lin Guo, Deyin Guo, Huabin Zhao, Yu Chen, Xiaolu Zhao

**Affiliations:** ^1^ State Key Laboratory of Virology and Biosafety, Modern Virology Research Center and RNA Institute, College of Life Sciences and Frontier Science Center for Immunology and Metabolism, Wuhan University, Wuhan, China; ^2^ Centre for Infection and Immunity Studies (CIIS), School of Medicine, Shenzhen Campus of Sun Yat-sen University, Guangzhou, China; ^3^ Hubei Key Laboratory of Cell Homeostasis, College of Life Sciences, Wuhan University, Wuhan, China; ^4^ Animal Biosafety Level-III Laboratory/Institute for Vaccine Research, Wuhan University, Wuhan, China; ^5^ Guangzhou Laboratory, Bio-island, Guangzhou, Guangdong, China

**Keywords:** SARS-CoV-2, mouse hepatitis virus, BMDCs, proteomics, transcriptomics, virus-host interaction, innate immunity

## Abstract

**Introduction:**

Understanding the intricacies of the host inflammatory response to coronaviruses is essential for developing effective therapeutic strategies to mitigate the severe consequences of these infections. Various coronaviruses can trigger the host immune response, leading to highly similar inflammatory reactions. The mouse hepatitis virus (MHV), which belongs to the same group of beta-coronaviruses as SARS-CoV-2 and induces high pathogenicity in mice, typically serves as a safety model for investigating highly pathogenic coronavirus infections, replication, and virus-host interactions.

**Methods:**

In this study, we conducted a comprehensive analysis of the transcriptome and proteome of mouse bone marrow dendritic cells (BMDCs) infected with MHV.

**Results:**

We characterized the global gene changes at both the mRNA and protein levels following viral infection, identifying ten genes involved in various anti-MHV biological processes. Furthermore, by integrating our findings with relevant published data on SARS-CoV-2 infection in cells, we observed significant similarities in the responses to MHV and SARS-CoV-2, particularly regarding immune and inflammatory responses.

**Discussion:**

These findings underscore how our research enhances the understanding of global gene expression alterations during coronavirus infection and facilitates the identification of novel antiviral targets.

## Introduction

In December 2019, the global outbreak of the viral illness COVID-19, caused by SARS-CoV-2 (1), garnered worldwide attention. As of April 22, 2024, SARS-CoV-2 has persisted for four years, resulting in over 775 million confirmed infections and nearly 7 million reported deaths worldwide (2). SARS-CoV-2 is a positive-sense, single-stranded RNA virus that belongs to the Beta-coronavirus genus part of the Coronaviridae family (3–5). Two other members of the Beta-coronavirus genus, the severe acute respiratory syndrome coronavirus (SARS-CoV) and the Middle East respiratory syndrome coronavirus (MERS-CoV), were responsible for outbreaks with significant fatality rates in 2002 and 2012, respectively (6, 7). To prepare for potential future outbreaks of coronavirus infections, it is essential to conduct research on the genes associated with these infections, particularly focusing on the common changes in gene expression that occur during such events. However, the stringent requirements for conducting research on SARS-CoV-2, SARS-CoV, and MERS-CoV necessitate the use of biosafety level 3 (BSL-3) laboratories (8, 9). Consequently, the murine hepatitis virus (MHV), which is also a member of the Beta-coronavirus genus and exhibits significant homology with SARS-CoV-2, SARS-CoV, and MERS-CoV, along with shared mechanisms and enzymes involved in genome expression, is frequently employed in this research domain. MHV serves as a safety model for investigating highly pathogenic coronavirus infections, their replication, and the interactions between the virus and its host (10, 11).

The innate immune system serves as the first line of defense against viruses, particularly during acute infections such as those caused by coronaviruses. However, the infection of most cell lines by coronaviruses is often insufficient to effectively activate antiviral innate immunity (12, 13). Instead, it does prompt the activation of cellular immunity and inflammatory responses in primary cells (14–17). Currently, SARS-CoV-2 infection models for human primary lung type 2 epithelial cells are not yet fully developed. Similar to SARS-CoV-2, MHV has been reported to infect the lungs, liver, brain, kidneys, and small intestine of mice, resulting in histopathological damage (18–20). Moreover, MHV can infect immune cells, including macrophages and dendritic cells, thereby effectively activating host cell immunity and inflammation. Previous studies have indicated that dendritic cells can activate type I interferon responses to coronaviruses (14, 15, 21, 22). Nonetheless, the specific characteristics of the immune and inflammatory responses, as well as the overall host immune cell expression profile following the infection of respective primary host cells by SARS-CoV-2 and MHV remain unclear. Therefore, the shared characteristics and commonalities of these two highly pathogenic coronavirus infections, which elicit whole-cell responses in the host—particularly regarding immune and inflammatory responses—warrant further investigation.

Transcriptomics is extensively utilized for analyzing samples infected with viruses, including SARS-CoV-2 (23–27). These investigations provide comprehensive insights into the impact of coronavirus infection on host transcriptomes, revealing distinct host inflammatory response signatures associated with coronavirus infection, including interferon response patterns and expression profiles of chemokines and cytokines. SARS-CoV-2 infection induces low-level expression of interferon response genes in host cells while triggering robust upregulation of chemokines and cytokines, potentially contributing to the cytokine storm observed in COVID-19 patients. In contrast, MHV infection also modulates host gene expression but exhibits differential alteration patterns compared to SARS-CoV-2. Enrichment analysis of differentially expressed genes from these transcriptomic studies demonstrated significant enrichment in pathways related to interferon signaling, inflammatory responses, apoptosis, and immune cell functionality. However, mRNA expression does not accurately reflect absolute or relative protein levels due to various RNA and protein modifications, as well as numerous dynamic regulatory processes involved in the translation of proteins from mRNAs. Factors such as mRNA stability, translation rate, protein product stability, post-translational modifications, and subcellular localization can all influence the results (28). The integration of mass spectrometry technology, antibody-based protein complex affinity purification, and protein crosslinking has been employed to systematically investigate proteome changes in virus-host protein interactions during viral infection (29–32). Transcriptome and proteome analyses exhibit a degree of complementarity. By combining quantitative proteomics with transcriptomics or genomics, researchers can achieve a comprehensive understanding of host-cell alterations following viral infection (33, 34).

In this study, we conducted a comprehensive analysis of the transcriptome and proteome of mouse bone marrow dendritic cells (BMDCs) infected with mouse hepatitis virus (MHV), a member of the beta-coronavirus group that includes SARS-CoV-2. We characterized the global gene expression changes at both the mRNA and protein levels following viral infection and identified ten genes involved in various biological processes that exhibit anti-MHV functions. Furthermore, we observed a significant similarity in the responses to MHV and SARS-CoV-2, particularly concerning immune and inflammatory responses. These findings indicate that our research contributes to a deeper understanding of the global gene expression alterations during coronavirus infections and aids in the identification of novel antiviral targets.

## Results

### Quantitative proteomic and transcriptomic analysis of BMDC infected with mouse hepatitis virus A59

**Figure 1:**
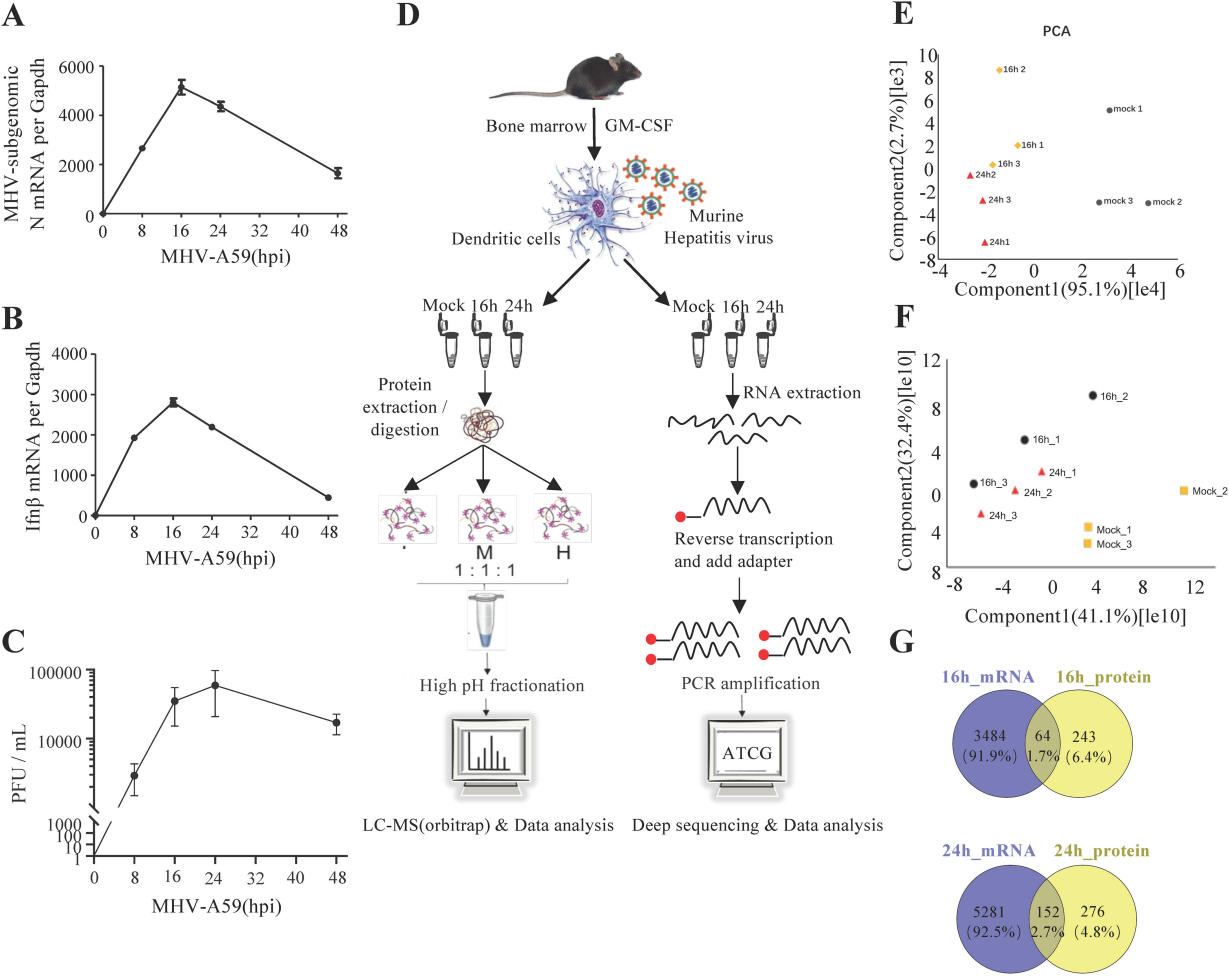
Mouse hepatitis virus can infect BMDCs and activate the innate immune response. **(A, B)** qRT-PCR analysis of *Ifnb* and *mRNA7* in MHV-infected BMDCs at different time points, as indicated. MHV mRNA7 is a subgenomic mRNA of the N gene. **(C)** Plaque assay of MHV-infected BMDC culture supernatants at different time points. **(D)** Schematic of the experiment. Bone marrow cells were isolated from mouse tibias and femurs and cultured for 7 to 9 days in a medium containing mouse GM-CSF. BMDCs were infected by MHV (MOI=1) and collected at 16 or 24 hpi. The cells were examined for both transcriptome and proteome analyses. Data are representative of three independent experiments (mean ± SD in **(B)**). **(E, F)** PCA of proteome **(E)** and transcriptome **(F)** data. **(G)** Venn diagrams showing the number of genes whose proteomics and transcriptomics changed together at 16 and 24 h.

We utilized MHV-A59-infected mouse BMDCs as our infection model to investigate the processes and mechanisms involved in host-coronavirus interactions during infection. BMDCs were derived from mouse bone marrow cells and stimulated with granulocyte-macrophage colony-stimulating factor (GM-CSF). The flow cytometric analysis revealed that the CD11c+ positive rate reached 80%, indicating that BMDC cells were successfully induced (**Supplementary Figure 1**). Initially, we established the appropriate time points for a time-course quantitative proteomic and transcriptomic study. We monitored the relative intracellular RNA levels of the MHV-N gene and *Ifnb* in BMDCs at different time points (**Figures 1A, B**). At 16 hours post-infection (hpi), viral replication (**Figure 1C**) and the innate immune response were observed to peak. By 24 hpi, host cell proteins had activated antiviral functions, resulting in the attenuation of the virus. Consequently, we concluded that 16 and 24 hpi should be designated as the two critical infection time points for our multi-omic analysis.

To investigate the intracellular changes following viral infection, we employed transcriptomics and proteomics to characterize the MHV infection of BMDCs at 0, 16, and 24 hpi (**Figure 1D**; **Supplementary Tables 1**, **2**). Using a threshold of ≥1 TPM (transcripts per million) to define detectable transcripts, our RNA-seq data identified 12659 genes, representing 62.2% of the protein-coding genome. Our label-free quantification workflow detected 4312 proteins at a false discovery rate (FDR) <1% with ≥2 unique peptides per protein, covering ~20% of the predicted proteome. Our results demonstrated that the quantification of the identified proteins and mRNAs was reproducible, as evidenced by the high correlation values between replicates (**Supplementary Figures 2A, B**). We compared the intensities of the quantified proteins and mRNAs to assess the degree of correlation between the replicates under different conditions. Cells were color-coded by intensity based on the Pearson coefficient values. Pearson coefficient values exceeding 0.8 in the quantitative proteomic and transcriptomic analyses indicated strong reproducibility between replicates and time points (**Supplementary Figures 2A, B**). Furthermore, we conducted principal component analysis (PCA) on the proteomic and transcriptomic data. (**Figures 1F, G**). Notably, we observed a greater degree of similarity between the two infection time points compared to the uninfected cells. A heatmap illustrating transcriptome and proteome regulation demonstrated significant differences in host-cell protein and mRNA levels following infection, while also revealing strong reproducibility between replicates and marked changes between conditions (**Supplementary Figures 2C, D**). The volcano plot of differential gene expression in RNA-seq was performed in **Supplementary Figure 3**. And few differentially expressed genes were validated by RT-qPCR in RNA-seq (**Supplementary Figure 4**). Our study identified 3548 significantly regulated genes from mock to 16 hpi and 5433 from mock to 24 hpi, as well as 307 significantly regulated proteins from mock to 16 hpi and 428 from mock to 24 hpi (**Figure 1E**). Importantly, we found that 216 genes exhibited the same degree of regulation at both the mRNA and protein levels, indicating consistency in gene transcription and translation. Most of the overlapping genes of transcriptome and proteome are co-upregulated or co-downregulated (**Supplementary Figure 5**). In contrast, other genes tended to change at either the mRNA or protein levels, as illustrated in **Figure 1E**.

### GO enrichment analysis of regulated proteins and protein-protein interaction networks

**Figure 2:**
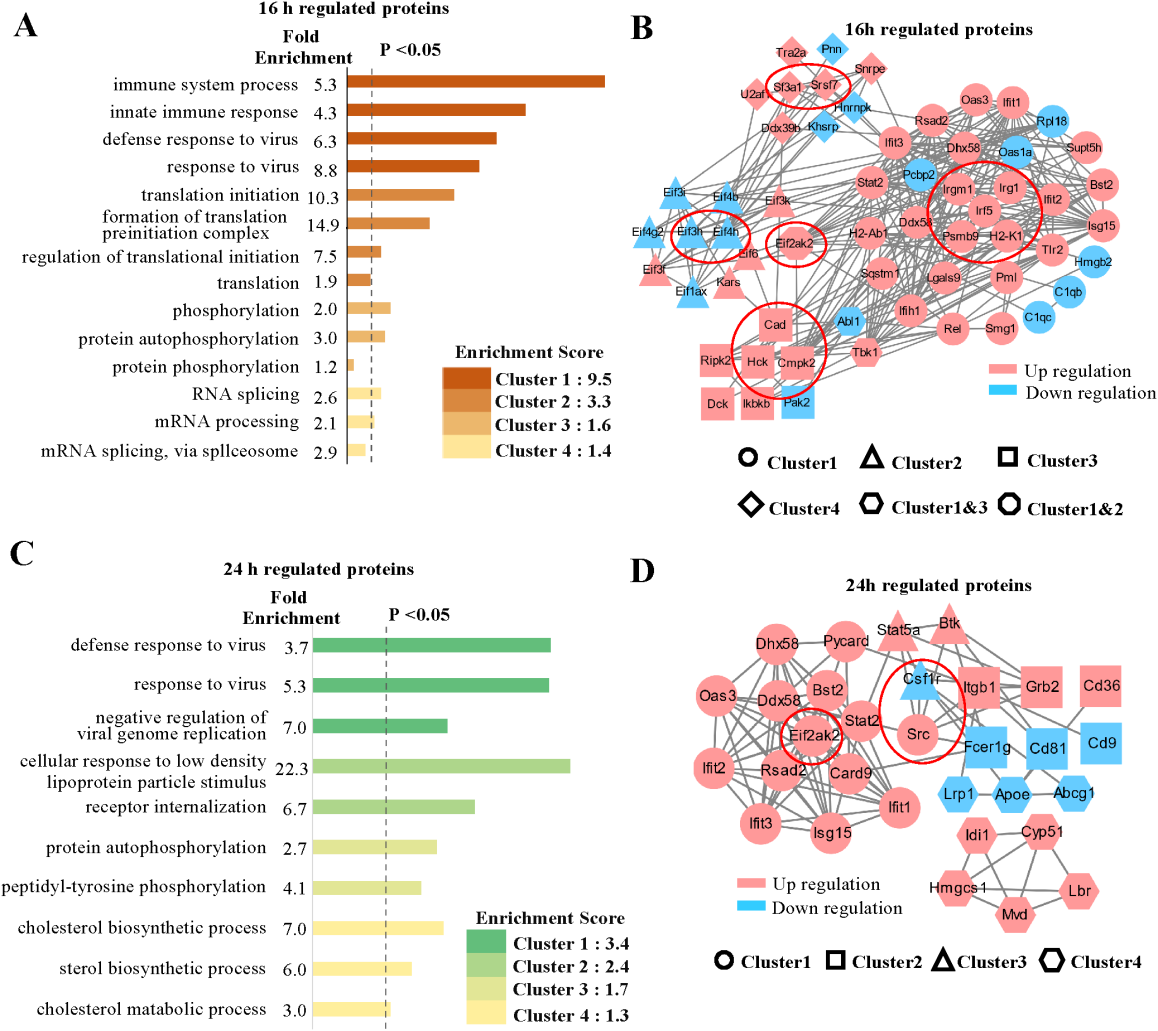
GO analysis and protein-protein interaction networks of regulated proteins. **(A, C)** GO cluster enrichment analysis of proteins regulated at 16 and 24 hpi, respectively, using DAVID software. The different enriched biological processes with distinct fold enrichment values were classified into four cluster groups according to their cluster enrichment scores and are listed in descending order by log (p value). Each cluster group contains multiple GO terms. The dotted line refers to P values less than 0.05. **(B, D)** Protein-protein interaction networks of the proteins in enriched clusters, **(A)** and **(C)** identified by the STRING analysis. Different graphic symbols represent different cluster groups. Red indicates upregulated proteins, and blue indicates downregulated proteins. The red circle indicates selected proteins of interest, high confidence (0.7).

We initially concentrated on the regulated proteins, particularly those exhibiting similar regulation at the mRNA level. Among the quantified host proteins, 172 were found to be upregulated and 135 downregulated at 16 hpi, while at 24 hpi, 318 were upregulated and 110 downregulated (**Figure 1E**). To gain insights into the alterations occurring in host cells during MHV-A59 infection, we conducted a bioinformatics functional enrichment cluster analysis of the regulated proteins. The DAVID software was utilized to identify enriched pathway clusters (35). Considering the distinct cellular immune responses observed at 16 and 24 hpi (**Figures 2A, C**), we analyzed the two time points separately. At 16 hpi, the regulated proteins were enriched in four primary clusters based on the unique cluster enrichment score (**Figure 2A**). The most enriched biological processes identified included “immune system processes”, “defensive response to viruses”, “translation”, “phosphorylation”, and “RNA splicing” (**Supplementary Table 3**). Additionally, we analyzed the RNA-Seq data and observed similar Gene Ontology (GO) enrichment patterns (**Supplementary Figure 6A**, **Supplementary Table 4**). To gain a comprehensive understanding of the protein-protein interactions among the regulated proteins in the four enriched clusters at 16 hours post-infection (hpi), we conducted a STRING analysis (36) (**Figure 2B**). Different graphical symbols were employed to represent the various clusters identified in the DAVID GO analysis, with red and blue indicating upregulation and downregulation, respectively. A higher percentage of proteins was found to be upregulated upon infection. Interactions among proteins within the same cluster were abundant, and notable interactions between clusters were also observed, particularly between Cluster 1 (immune and defensive response to viruses) and Cluster 2 (translation), as well as between Clusters 1 and 4 (RNA splicing).

The GO enrichment analysis of the regulated proteins at 24 hpi revealed several enriched cellular processes that were also identified at 16 hpi; however, distinct enriched biological processes were additionally found (**Figure 2C**). In particular, alongside processes such as “defense to virus” and “phosphorylation”, we noted a significant number of proteins associated with “metabolic processes”, particularly in cholesterol metabolism (**Supplementary Table 5**). We conducted a similar analysis on the 24 hpi transcriptome data, which yielded comparable GO enrichment patterns (**Supplementary Figure 6B**, **Supplementary Table 4**). Furthermore, we examined the protein-protein interaction network of the proteins involved in the enriched processes at 24 hpi using STRING analysis (**Figure 2D**). As observed at 16 hours post-infection (hpi), a greater number of proteins were found to be upregulated than downregulated, indicating an active response to viral infection. Proteins in Cluster 1 were primary responsible for the majority of interactions related to immune defense, with their expression levels increasing at 24 hpi. Some proteins were also identified in the protein-protein network at 16 hpi, while others became active by 24 hpi (**Figure 2D**). Notably, interactions between Cluster 1 (defense to virus) and Cluster 2 (receptor and signaling pathway), as well as between Clusters 2 and 3 (phosphorylation), were particularly abundant. In contrast, protein interactions within Cluster 4 (metabolic process) exhibited a high degree of independence compared to those in other clusters, with proteins involved in cholesterol metabolism, such as Hmgcs1, being upregulated. The regulation of metabolic processes to maintain cellular homeostasis in response to viral infection is of significant interest and warrants further investigation.

**Figure 3:**
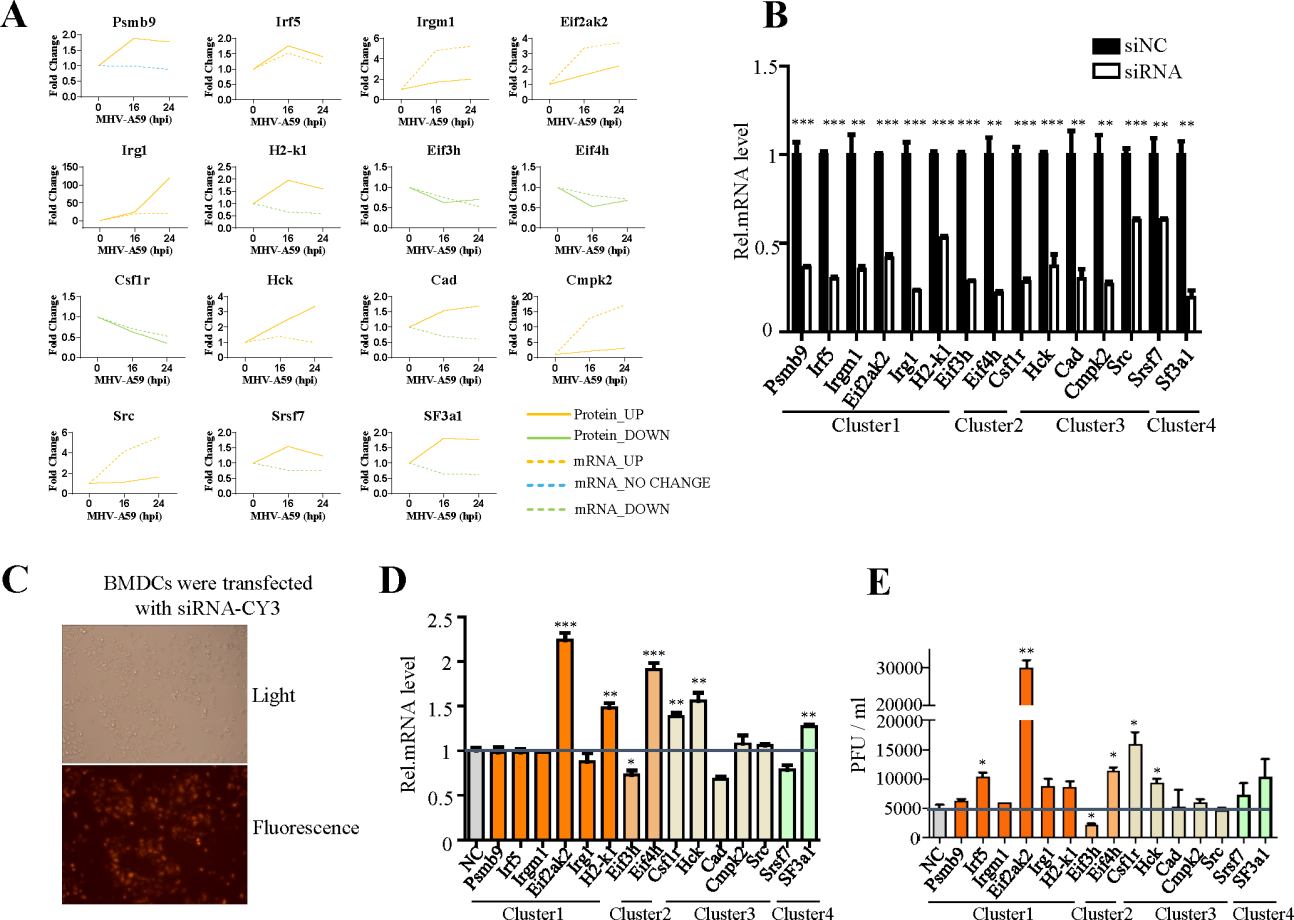
Verification of the antiviral function of selected genes. **(A)** Changes in mRNA (dotted line) and protein (solid line) levels of host proteins selected from **Figures 2B, D**. The vertical axis expresses the relative expression of mRNA or protein respectively. **(B)** qRT-PCR analysis to evaluate the efficiency of siRNAs. BMDC cells were transfected with negative control siRNA (si-NC) or target siRNAs (si-psmb9, si-Irf5, si-Irgm1, si-Eif3h, si-Eif4h, si-Eif2ak2, si-Irg1, si-Csf1r, si-Hck, si-Cad, si-Sf3a1, si-Cmpk2, si-Srdf7, si-Src, and si-H2-K1). The cells were collected 24 h post-transfection and subjected to qRT-PCR. **(C)** Cy3-labeled siRNA was transfected into BMDC cells, and the transfection efficiency was detected by fluorescence microscopy. **(D)** qRT-PCR analysis of mRNA7 in BMDCs transfected with target siRNA with siNC as the negative control. The cells were infected with MHV for 16 h, as indicated. **(E)** BMDCs were transfected with target siRNA with siNC as the negative control. The cells were infected with MHV for 16 h, as indicated. Culture supernatants were collected and used for plaque assays on L2 cells to determine the MHV titers. *P < 0.05, **P < 0.01, and ***P < 0.001. Data are representative of three independent experiments (mean ± SD in **(C)**).

To identify potential antiviral proteins, we selected specific proteins for further validation based on GO enrichment and protein-protein interaction analysis. The proteins (Psmb9, Irf5, Irgm1, Eif3h, Eif4h, Eif2ak2, Irg1, Csf1r, Hck, Cad, Sf3a1, Cmpk2, Srsf7, Src, and H2-K1) were located at the core of the interaction networks and exhibited strong interactions with proteins both within the same functional group and from other groups. We hypothesize that these proteins may play critical roles in antiviral defense. Our multi-omic analysis (**Figure 3A**) confirmed the changes in these proteins at both the mRNA and protein levels through quantification. Most genes demonstrated consistent regulation at the mRNA and protein levels. Notably, the expression levels of the majority of these proteins increased following viral infection, with the exception of three proteins (Eif3h, Eif4h, and Csf1r), which exhibited downregulation.

To investigate the antiviral activity of these genes during MHV infection, we designed siRNAs specifically targeting them (**Figure 3B**). The efficiency of siRNA transfection was evaluated by using small RNAs labeled with CY3 (**Figure 3C**). Subsequently, cells were infected at a multiplicity of infection (MOI) of 1, and both the cell supernatant and RNA were collected to determine the virus titer (**Figures 3D, E**). Our screening results indicated that Eif2ak2, H2-K1, Csf1r and Eif4h exhibit certain antiviral effects, while Eif3h appears to facilitate viral replication, as evidenced by qPCR and plaque assay (**Figures 3D, E**). To strengthen siRNA specificity, key genes showing significant impacts on viral replication in siRNA knockdown experiments (**Figures 3C, D**) were further validated using CRISPR-Cas9-mediated gene knockout. The concordant results (**Supplementary Figure 9**) strongly support the siRNA-specific effects rather than off-target artifacts.

**Figure 4:**
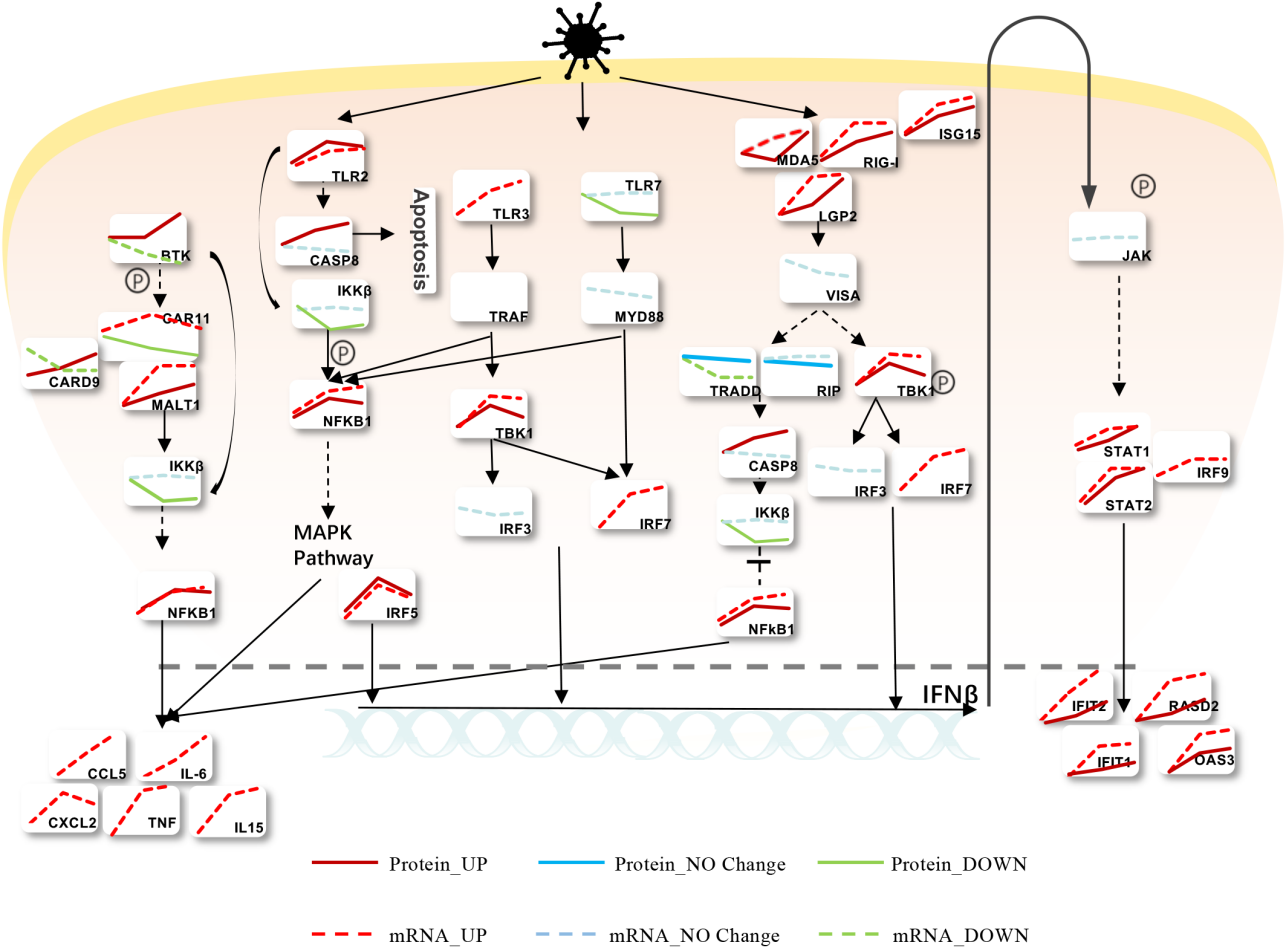
Modulation of Intracellular Signaling Pathways during MHV Infection. Infected BMDCs were infected with MHV, and we found that the cells mainly recognized the virus through TLR and RLR, activating IFNβ and the inflammatory response. Red means upregulated, while green means downregulated. Solid lines express protein levels, and sequences express mRNA levels.

Upon viral infection, cells initiate a rapid immune response, activating various immune and inflammatory signaling pathways. To elucidate the expression changes at both mRNA and protein levels for key genes involved in these pathways, we constructed a comprehensive map based on our transcriptomic and proteomic analyses of MHV-infected BMDCs. As shown in **Figure 4**, MHV-A59 activates Toll-like receptor (TLR) and RIG-I-like receptor (RLR) signaling pathways, suggesting their potential involvement in viral recognition. Notably, major receptors such as Mda5, Rig-I, and TLR2 exhibited upregulation at both the mRNA and protein levels. In contrast, while TLR3 levels increased at the mRNA level, it was not detectable at the protein level. Additionally, TLR7 was found to be downregulated at the protein level. Following infection, viral RNA is recognized by RLRs and TLRs, leading to modifications by downstream signaling proteins such as Visa, Tbk1, and Myd88. This cascade results in the phosphorylation of various adaptors that activate downstream transcription factors IRF7 and IRF3 within the nucleus, subsequently inducing the expression of type I interferon (IFNβ). The extracellular secretion of IFN-β activates the JAK-STAT pathway, promoting the production of numerous downstream interferon-stimulated genes (ISGs), including Ifit1, Ifit2, Rasd2, and Oas3. Notably, the mRNA and protein levels of these genes significantly increase following viral infection, with both Ifit2 and Rasd2 exhibiting upregulation across various coronavirus infections. Similarly, the NF-κB signaling pathway is activated through multiple routes upon the recognition of viral RNA by the receptors, leading to the upregulation of Nfkb1 at both mRNA and protein levels, and the subsequent production of several inflammation-related proteins, including Ccl5, Il-6, Cxcl2, Tnf, and Il-15. These inflammatory factors have been shown to be highly expressed following various types of coronavirus infections. This overview provides valuable insights into the changes in mRNA and protein levels of various genes associated with MHV-infected BMDCs.

### Global view of host proteins/mRNAs regulated in MHV-infected BMDCs

**Figure 5:**
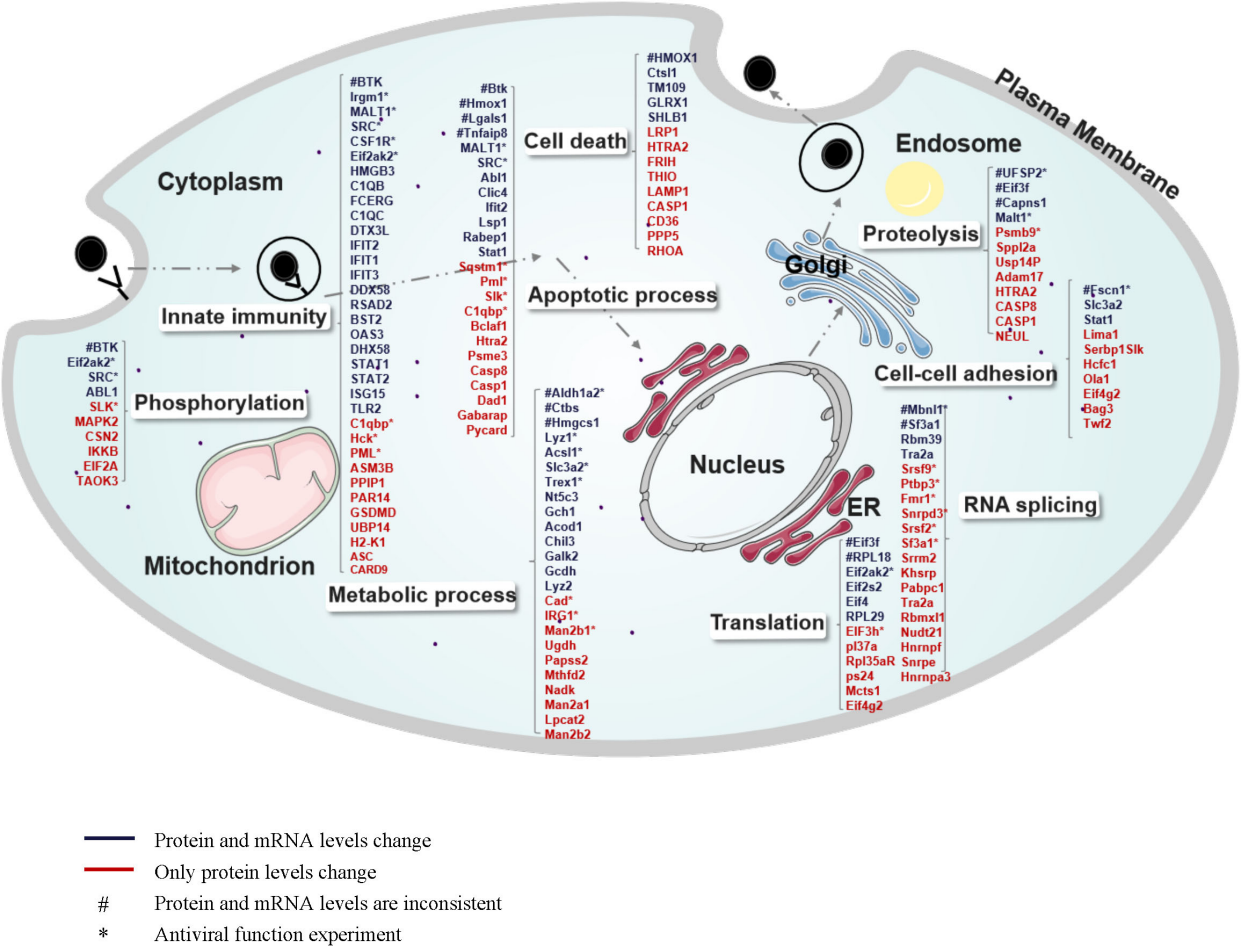
Global view of host proteins/genes regulated upon MHV infection of BMDCs. The regulated proteins/genes at 24 hpi are illustrated in different categories according to cellular components or biological processes annotated by GO analysis. Numerous proteins are involved in innate immunity, apoptotic processes, metabolic processes, phosphorylation, cell death, proteolysis, cell-cell adhesion, RNA splicing, and translation. Blue indicates consistent regulation at both the protein and mRNA levels, and red indicates regulation at the protein level only. “#” indicates inconsistent regulation at the mRNA level and protein level. “*” indicates that its antiviral function has been tested.

**Figure 6:**
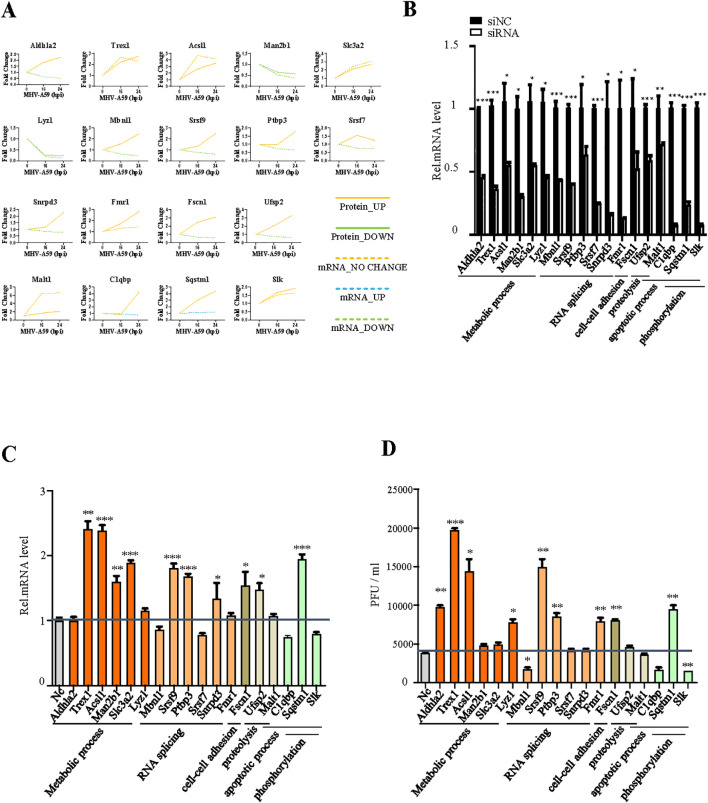
Verification of the antiviral function of selected genes. **(A)** Changes in mRNA (dotted line) and protein (solid line) levels of host proteins selected from **Figure 5**. The vertical axis expresses the relative expression of mRNA or protein respectively. **(B)** qRT-PCR analysis to evaluate the efficiency of siRNAs. BMDCs were transfected with negative-control siRNA (si-NC) or target siRNAs (si-Mbnl1, si-Srsf9, si-Usf2, si-Ptbp3, si-Sqstm1, si-Srsf2, si-Fscn1, si-Aldhla2, si-Trex1, si-Acsl1, si-Man2b1, si-Lyz1, si-Slc3a2, si-Malt1, si-Snrpd3, si-Fmr1, si-C1qbP, and si-Slk). The cells were collected 24 h post-transfection and subjected to qRT-PCR. **(C)** qRT-PCR analysis of mRNA7 in BMDCs transfected with target siRNA with siNC as the negative control. The cells were infected with MHV for 16 h, as indicated. **(D)** BMDCs were transfected with target siRNA with siNC as the negative control. The cells were infected with MHV for 16 h, as indicated. Culture supernatants were collected and used for plaque assays on L2 cells to determine the MHV titers. *P < 0.05, **P < 0.01, and ***P < 0.001. Data are representative of three independent experiments (mean ± SD in **(C)**).

To gain a comprehensive understanding of how MHV infection influences the biological processes of host cells, we developed a global cellular response map. In this map, the regulated proteins and genes were categorized based on their GO functions (**Figure 5**). Due to the increased regulation of proteins and the involvement of a greater variety of biological processes at 24 hours post-infection (hpi) compared to 16 hpi, we illustrate our proteomics and transcriptomics data collected at 24 hpi. As shown in **Figure 5**, numerous biological processes and protein complexes are evidently regulated as a consequence of MHV infection. Viral attacks not only elicit a robust innate immune response in cells but also induce alterations across a wide range of cellular processes, including metabolism, transcription, translation, protein hydrolysis, and apoptosis, among others. We propose that the innate immune response is not the sole mechanism for combating the virus; our findings indicate that genes previously deemed unrelated to innate immunity also exhibit antiviral functions (**Figures 3D, E**). Host cells actively respond by regulating various cellular functions, thereby maintaining cellular homeostasis throughout the entire infectious life cycle, from the virus’s entry for replication to its eventual release. As indicated in **Figure 5**, the majority of genes involved in diverse cellular processes displayed similar trends of alteration at both the mRNA and protein levels. Blue denotes consistent changes at both the protein and mRNA levels, while red indicates regulations observed solely at the protein level. A few genes exhibiting inconsistent regulation between the protein and mRNA levels are marked with #. The antiviral functions of genes marked with * have been tested. (**Figures 3**, **6**).

We selected 18 genes involved in various biological processes, including metabolic processes, RNA splicing, cell-cell adhesion, proteolysis, apoptotic processes, and phosphorylation and compared their alterations at the mRNA and protein levels using several biological assays (**Figure 6A**). Six genes exhibited consistent changes at both the mRNA and protein levels: Trex1, Acsl1, Slc3a2, Fmr1, Malt1, and Slk. Among these, Trex1, Acsl1, and Slc3a2 are specifically associated with metabolic processes. The changes observed in the mRNA and protein levels of the other genes differed, which may reflect the dynamic regulation occurring between intracellular mRNAs and proteins. Subsequently, we designed siRNAs targeting these 18 genes to investigate their antiviral effects in MHV infection (**Figure 6B**). According to qPCR analysis and plaque assays, Trex1, Acsl1, Srsf9, Ptbp3, Sqstm1 and Fscn1 demonstrated antiviral effects, while Mbnl1, C1qbp, and Slk appeared to promote viral replication (**Figures 6C, D**). These altered proteins play significant roles in metabolic processes, RNA splicing, and apoptosis, indicating that these biological processes may serve as potential targets for antiviral response. To confirm the specificity of these findings, CRISPR/Cas9-mediated knockout validation was performed on key genes exhibiting significant effects on viral replication in siRNA screening assays (**Figures 6C, D**). The consistent results (**Supplementary Figure 9**) robustly corroborated the identical effects on the virus following the knockdown of target genes.

### Analysis of co-varying factors in coronavirus infections

**Figure 7:**
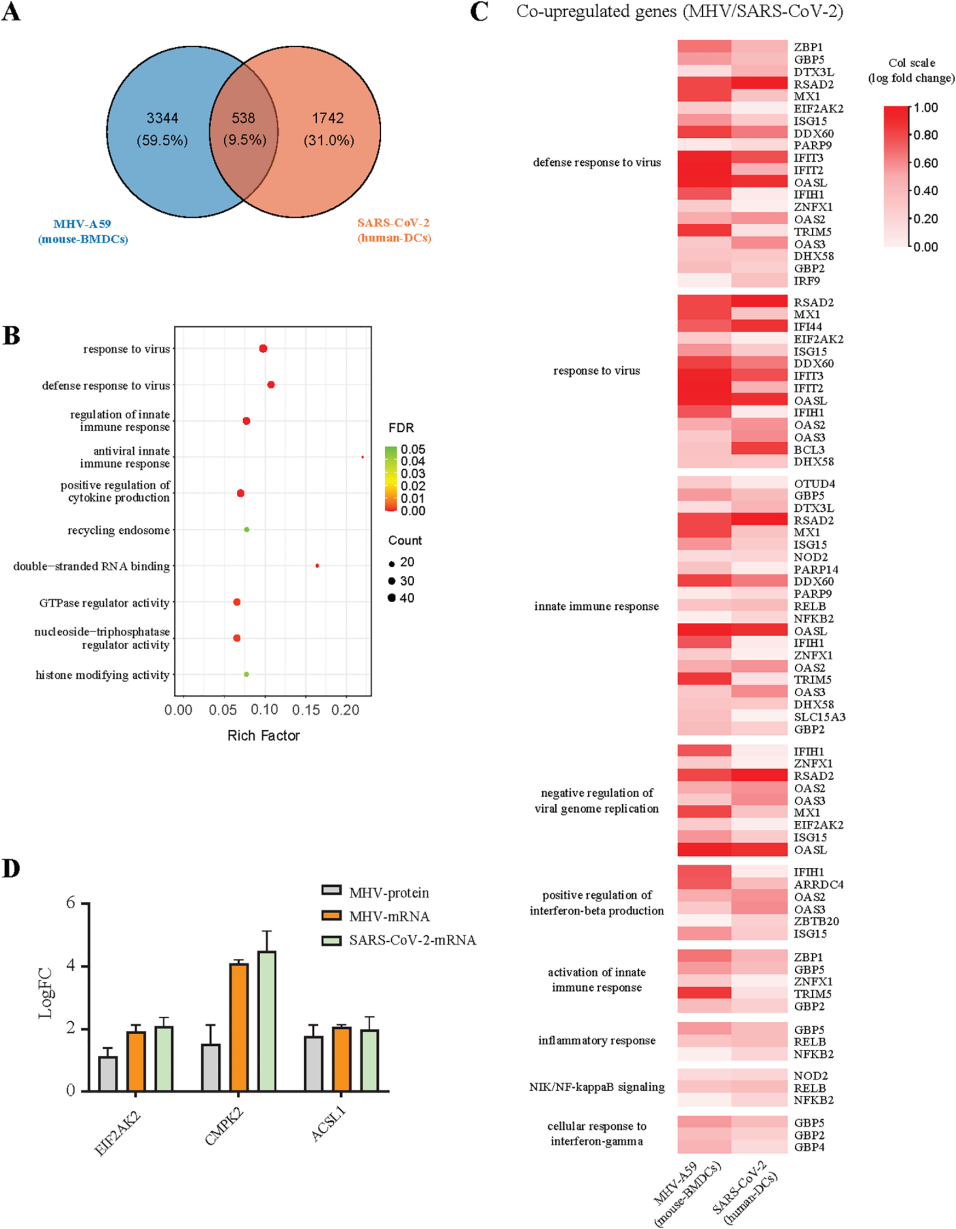
Analysis of co-variation factors between SARS-CoV-2 and MHV infections. **(A)** The Venn diagram shows the number of genes that undergo simultaneous changes in two datasets: MHV-infected BMDCs, SARS-CoV-2-infected human DCs. **(B)** KEGG pathway enrichment analysis of genes co-upregulated together in two databases. **(C)** The heatmaps show the expression levels for MHV-A59 and SARS-CoV-2 demonstrating the co-upregulation of genes related to immunity and inflammation, the log fold change values of the data were normalized using a column scaling (col scale) method. **(D)** The expression levels of antiviral genes (EIF2AK2, CMPK2, and ACSL1) in MHV and SARS-CoV-2 infected cells.

After conducting a multi-omics analysis on MHV, we sought to integrate this data with that of COVID-19 infection for a joint analysis, with the objective of identifying co-regulated genes in MHV-A59-infected BMDCs and SARS-CoV-2-infected cells. We obtained relevant data pertaining human DCs from COVID-19 patients, as documented in previous studies (23, 24, 37). A variety of genes exhibited similar regulation across different viral infection conditions. We conducted a GO enrichment analysis of the co-regulated genes (**Figures 7A, B**, **Supplementary Figure 6**). The results indicated that 538 genes were up-regulated across the three types of viral infections, primarily involved in novel pathways related to antiviral responses and innate immunity. Among these co-varying genes, three genes (Eif2ak2, Cmpk2, and Acsl1) mentioned earlier were specifically examined for their antiviral functions (**Figures 3**, **6**, **7C**). Among the identified genes, Eif2ak2 and Acsl1 exhibited significant antiviral activity against MHV. Our focus was specifically on genes that co-vary with antiviral and innate immune responses, several of which have been demonstrated to be associated with coronavirus infection and replication, including EIF2AK2, MX1, and ISG15 (38–40) (**Figure 7D**). Consequently, we contend that investigating these commonly varying genes is instrumental for a deeper understanding of the mechanisms underlying coronavirus infection and antiviral research.

Interestingly, we identified several characteristics of gene expression following SARS-CoV-2 infection. We selected genes that exhibited significant changes (log2 fold change > 1) after SARS-CoV-2 infection in Calu3 and human dendritic cells (DCs), while showing minimal changes (fold change < 0.5) after MHV infection, and conducted a GO enrichment analysis on these genes. The results indicate that a total of 89 genes were upregulated (**Supplementary Table 8**). Notably, among the uniquely upregulated genes, Il8, Ccl20, Casp1, Csf3, Irf2, Trim14, Tnfrsf25, and Jak2 are implicated in immune and inflammatory signaling pathways, which may contribute to the hyperinflammatory response associated with SARS-CoV-2. Furthermore, the functions of many of these genes remain unannotated, suggesting the potential discovery of novel functional genes.

## Discussion

To address both current and potential future outbreaks of coronavirus, it is essential to deepen our understanding of the mechanisms underlying coronavirus infection. Investigating the commonalities among various coronavirus infections may aid in identifying new broad-spectrum antiviral targets. However, the study of highly pathogenic coronaviruses, particularly the SARS-CoV-2 infection model, presents significant limitations. Due to the highly pathogenic nature of SARS-CoV-2, all infection experiments must be conducted in biosafety level 3 laboratories or higher. The costs associated with such laboratories and the use of hACE2 mouse models are substantial, thereby greatly restricting research on SARS-CoV-2. Although alternative research methods, such as pseudoviruses and replicons, exist, these approaches cannot fully replicate the entire infection cycle of the virus. Our research group has long been engaged in studies involving MHV. (41–45). MHV is similar to SARS-CoV-2 and belongs to the beta-coronavirus genus. With the exception of the different hosts, the entire viral infection cycle is analogous to that of SARS-CoV-2. Additionally, the infected mouse model is well-established and can develop pneumonia and hepatitis through nasal drip infection and *in situ* liver injection. Following infection, elevated levels of inflammatory factors are produced in the lungs and liver, leading to tissue damage. Furthermore, only a biosafety level 2 laboratory is required to conduct these experiments, making this model both mature and cost-effective. Transcriptome and proteome analyses conducted 16 and 24 hours after MHV infection of BMDCs revealed the activation of signaling pathways associated with “immune system processes” and “defensive response to viruses” (**Figure 2**). Moreover, an integrated analysis of omics data from SARS-CoV-2 infections indicated that the genes exhibiting changes show some similarities and are concentrated in signaling pathways related to antiviral innate immunity (**Figure 7**). Our MHV-infected BMDCs model serves as an ideal viral model for investigating the relationship between coronaviruses and the host’s innate immune response.

We utilized the MHV-infected BMDC model to collect cell samples at 0, 16, and 24 hpi to investigate the dynamic changes that occur following viral infection, including various biological processes and signaling pathways. Although transcriptomics encompasses a broader range of genes compared to proteomics, the GO analysis of the two datasets revealed significant similarities. This gene coverage is consistent with technical limitations of deep proteome profiling in complex samples. RNA-seq is a high-throughput sequencing technology that can generate a large amount of data in a single run. It can sequence millions of short reads, allowing for the detection of a wide range of transcripts with different expression levels. RNA-seq has a wide dynamic range, which means it can accurately quantify gene expression levels from very low to very high. The proteome is much more complex than the transcriptome. Proteins have a wide range of molecular weights, isoelectric points, and post-translational modifications. These factors make it difficult to cover the entire proteome using MS/MS. The complexity of the proteome also leads to ion suppression and other issues during the MS/MS analysis, which can affect the detection of low-abundance proteins. Notably, at both 16 and 24 hpi, the analyses indicated comparable cell enrichment pathways (**Figure 2**; **Supplementary Figure 6**, **Supplementary Tables 3**-**5**). This presents a valuable resource for further exploration of gene functions at the mRNA and protein levels concerning host-virus interactions. As anticipated, biological pathways related to immune processes and the defensive response to the virus were the most significantly enriched at 16 hpi. The proteins involved in these pathways primarily function in virus recognition and the activation of the immune system, thereby triggering the cellular innate immune response and host defense mechanisms against the virus through a series of cascades. In addition to immune-related processes, proteins associated with RNA splicing, translation initiation, and phosphorylation were also significantly enriched at 16 hpi. This findings suggests that host cells adapt their protein synthesis and other functional switches in response to the stress induced by viral infection, transitioning from the mock condition to 16 hpi. A similar analysis conducted on the 24 hpi data revealed comparable GO enrichment patterns, indicating that, by this time, host cells enter another phase characterized by metabolic changes due to viral infection. At 16 hpi, the innate immune response was found to be predominant. The recognition of viral RNA by cellular receptors activates immune stress signaling pathways through a series of phosphorylation events, generating various interference mechanisms via transcription and translation, thereby enabling cells to combat viruses and regulate overall cellular responses. By 24 hpi, the innate immune response appears to be partially supplanted by proteins with antiviral functions, as an excessive immune response may lead to cellular damage. Consequently, proteins involved in cellular metabolism undergo changes, suggesting that genes associated with cell survival may facilitate the fight against the virus through specific mechanisms.

Our research identified 64 genes at 16 hours and 152 genes at 24 hours. A total of 216 genes exhibited consistent changes at both the mRNA and protein levels (**Figure 1E**), indicating a correlation between transcription and translation for these genes. Among those verified to possess antiviral effects, the protein levels of most aligned with the changes observed in mRNA levels (such as Eif2ak2, Trex1, Acsl1, Csf1r, Hck, and Eif4h), suggesting that genes with concurrent changes in transcription and translation are particularly relevant to this study. To enhance the specificity of siRNA, we further validated key genes that exhibited substantial influences on viral replication in siRNA knockdown experiments (**Figures 3**, **6**) through CRISPR-Cas9-mediated gene knockout. The consistent findings (**Supplementary Figure 9**) robustly corroborate the specific effects of siRNA rather than off-target artifacts. Eif2ak2 has been reported to be as a potential antiviral target, Eif2ak2 inhibitor compound is a potent antiviral that could combat SARS-CoV-2 infection (46). Which also reveals that there are high similarities in the interactions of coronaviruses within host cells, and it is of great significance to study the impact of MHV on the host cell transcriptome level. Eif2ak2 exhibits a wide range of antiviral activity and is capable of enhancing the production of type-1 interferons through the activation of the integrated stress response (47). H2-K1 is a member of the mouse major histocompatibility complex (MHC) class I molecules and is homologous to the human HLA-A molecule. It plays a crucial role in the antigen presentation process, which allows the immune system to recognize and respond to foreign substances, such as pathogens (48, 49). During viral infections, Type I interferons activate multiple signaling pathways, including the JAK-STAT pathway. These pathways enhance the cell’s resistance to the virus while promoting the expression of MHC Class I molecules, such as H2-K1. This function is essential for the ability of immune cells, including BMDCs, to perform their immune functions. In the context of viral infection, the activation of Csf1r signaling can promote the proliferation and activation of macrophages, enhance their capacity to phagocytose and digest viruses, and play a crucial role in the antiviral immune response (50). Additionally, type II interferon, IFN-γ, can further promote macrophage activation by increasing the expression of Csf1r or modulating its downstream signaling pathways, thereby strengthening their ability to phagocytose and digest pathogens. Eif4h plays a crucial role in the initial stages of protein synthesis and may be involved in the regulation of viral replication and translation. During herpes simplex virus (HSV) infection, the virus-encoded virion host shutoff protein interacts with Eif4h, influencing the stability and degradation of mRNA (51). During viral infections, cells regulate protein translation via the interferon signaling pathway to combat viral invasion. As a translation initiation factor, Eif4h may play a role in this process by modulating the translation efficiency of mRNA in response to viral infection. This interaction could enable the virus to inhibit the synthesis of host cell proteins while simultaneously promoting the production of its own proteins. However, we also observed that a significant proportion of genes displayed divergent trends between mRNA and protein levels. This inconsistency is common and may arise from post-transcriptional and post-translational modifications of the proteins. Notably, among the genes confirmed to have antiviral effects, the protein and mRNA levels of Srsf9, Sqstm1, Ptbp3, and Fscn1 exhibited differing changes. While further research is necessary to elucidate the underlying mechanisms, the discrepancies in protein and mRNA levels following viral infection underscore the importance of dual-omics approaches to identify valuable antiviral factors. Previous reports (52, 53) on the transcriptomic changes induced by MHV infection in host cells have demonstrated that MHV can regulate the expression of a diverse array of genes, many of which are directly linked to innate and acquired immune responses. This finding aligns with our research. Furthermore, the reproducibility of these results suggests that MHV serves as an effective model virus for studying the inflammatory responses elicited by coronaviruses. We further compared our findings with previous studies (40) on MHV-infected murine primary immune cells PMs and BMDMs in **Supplementary Figure 8**. The comparative analysis revealed 314 overlapping genes across the three cell types’ RNA-seq datasets, with 78.4% of these genes demonstrating consistent upregulation or downregulation patterns. These shared differentially expressed genes were primarily enriched in critical biological processes including immune signaling pathways, cellular responses to viral infection, activation of innate immunity, and inflammatory responses. Furthermore, pathway analysis identified significant involvement of defense response to virus and innate immune response. The substantial concordance in transcriptional regulation across different immune cell populations suggests conserved mechanistic responses to MHV infection at the molecular level. These findings collectively indicate that murine immune cells maintain fundamental similarities in both genetic regulation and functional adaptation when confronting MHV challenge, particularly in their core antiviral defense mechanisms and immunoregulatory networks. Unlike MHV and other coronaviruses, SARS-CoV-2 uniquely suppresses innate immune responses. At low multiplicities of infection, SARS-CoV-2 minimally induces IFN-I/III production yet robustly triggers chemokine expression (23). Notably, SARS-CoV-2 does not directly infect PBMCs but indirectly induces lymphocyte apoptosis via cytokine storm mechanisms (24). SARS-CoV-2 dysregulates interferon signaling while hyperactivating cytokines. Similar to MHV, it exploits integrins for entry and immune evasion. Complementing these findings, Islam et al. (26) compared transcriptional responses across infection models and demonstrated compartmentalized host reactions. Nasopharyngeal samples showed activation of innate immunity and interferon signaling, but paradoxically suppressed apoptosis and antigen presentation pathways. In contrast, lung tissues exhibited hyperactivation of cytokine signaling and integrin-related genes, potentially facilitating viral entry and inflammatory damage. Strikingly, SARS-CoV-2 non-structural proteins were computationally predicted to interact with host factors regulating integrin signaling and immune evasion, aligning with observed upregulation of these pathways in severe COVID-19.

We identified a series of novel antiviral genes using a multi-omic approach. In addition to genes with established antiviral functions that are implicated in the immune response, we discovered that genes associated with other activated cellular processes—such as metabolic processes (Trex1, Acsl1, Man2b1, Slc3a2, and Aldhla2), phosphorylation (Csf1r, Hck, and Sqstm1), RNA splicing (SF3a1, Srsf9, and Ptbp3), and translation (Eif4h)—also exhibit varying levels of antiviral activity. On one hand, these genes possess previously unrecognized antiviral functions. On the other hand, they contribute to biological processes that facilitate their antiviral roles, as viral infections of cells represent events that engage the entire cell, leading to alterations in overall gene mRNA and protein levels (**Figures 3**, **6**).

The utilization of MHV-A59 as a model for studying coronavirus-host interactions offers distinct advantages, particularly in the context of biosafety and experimental tractability. As a member of the Beta-coronavirus genus, MHV-A59 shares conserved genomic structure, replication strategies, and structural features with SARS-CoV-2 and other coronaviruses, enabling investigations into fundamental mechanisms of coronavirus biology (3, 54). The inability of MHV-A59 to infect humans, combined with its well-established utility in murine systems, facilitates mechanistic studies in immune cells such as BMDCs without the stringent biosafety constraints associated with SARS-CoV-2. Furthermore, prior work has highlighted conserved molecular processes, such as the formation of double-membrane vesicles and viral RNA export channels, which are critical for coronavirus replication and are shared between MHV and SARS-CoV-2 (55, 56). These parallels underscore the value of MHV-A59 as a tool for probing conserved host-pathogen interactions and identifying potential pan-coronaviral therapeutic targets. However, direct extrapolation of findings from MHV-A59-infected BMDCs to SARS-CoV-2-infected human DCs requires careful consideration of key limitations. Notably, the two viruses exhibit divergent viral tropism and entry mechanisms: SARS-CoV-2 relies on hACE2 receptors, predominantly expressed in respiratory epithelia, whereas MHV-A59 utilizes CEACAM1, which is absent in SARS-CoV-2 infection (57, 58). Additionally, the contrasting dynamics of IFN-I responses—robust and rapid IFNβ induction by MHV-A59 versus delayed IFN-I suppression by SARS-CoV-2—highlight fundamental differences in immune evasion strategies (59, 60). These disparities suggest that while MHV-A59 infection models may elucidate conserved inflammatory pathways, they may not fully recapitulate the immunopathological features of SARS-CoV-2, such as cytokine storm dynamics.

Future studies should aim to integrate comparative datasets from SARS-CoV-2-infected human DCs to validate conserved mechanisms and contextualize model-specific discrepancies. Combining multi-omics approaches with targeted functional assays could further dissect shared versus virus-specific host responses. Additionally, exploring MHV-A59 in conjunction with other Beta-coronaviruses may refine its utility as a surrogate system. Ultimately, while MHV-A59 provides a pragmatic platform for probing coronavirus biology, particularly in studying conserved intracellular immune responses post-entry, its limitations underscore the necessity of complementary models to fully unravel the complexities of SARS-CoV-2 pathogenesis and inform therapeutic development.

## Materials and methods

### Ethics statement

All mice were housed in a specific pathogen-free animal facility at Wuhan University, and all animal experiments were conducted in accordance with the Chinese National Laboratory Animal Guidelines for the Ethical Review of Animal Welfare and approved by the Institutional Animal Care and Use Committee of Wuhan University (NO.15060C). The mice were euthanized using with CO_2_ prior to the various studies.

### Cells

Neuro 2a (ATCC Number: CCL-131) and L2 (ATCC Number: CCL-149) cells were obtained from the American Tissue Culture Collection. The cells were maintained in Dulbecco’s modified Eagle medium (DMEM) supplemented with 10% fetal bovine serum (FBS) (Gibco) and 1% penicillin-streptomycin (Gibco).

### Virus

Murine hepatitis virus (MHV) strain A59 (ATCC Number: VR-764), which is a positive single-stranded RNA (+ssRNA) virus that belongs to the *Beta coronavirus* genus in *Nidovirales*, which includes Middle East respiratory syndrome coronavirus (MERS-CoV) and severe acute respiratory syndrome coronavirus (SARS-CoV), was used as described previously (41–43). We utilized Neuro 2a cells specifically for the amplification of MHV to ensure a sufficient viral titer for subsequent experiments.

### Reagents

Sequencing Grade Modified Trypsin (Promega, Cat# V5280), DTT (Thermo Scientific, Cat# 21401), Iodoacetamide (Sigma-Aldrich, Cat# I1140), Dimethyl Labeling Reagents: Light (CH2O), Medium (CD2O), and Heavy (13CD2O) formaldehyde (Sigma-Aldrich, Cat# 42677, 42678, 42679 respectively), TRIzol Reagent (Invitrogen, #15596026), PrimeScript™ RT Reagent Kit (Takara, Cat# RR037A), Magnetic Beads with Oligo (dT) (Thermo Fisher Scientific, Cat# AM1802), SYBR Green Fast qPCR MasterMix (Yeasen, Cat# 11201-11203*).

### Plaque assay

Serial 10-fold dilutions of MHV were added to monolayer L2 cells seeded in 6-well plates. After 1 hour of adsorption at 37 °C, the inoculum was removed, and the cells were washed three times with PBS and then supplemented with DMEM containing 1.0% methylcellulose and 5% FBS. The plates were incubated for 2 days until obvious plaques were observed. The monolayers were stained with DMEM containing 0.5% neutral red for 4 h at room temperature, and the plaques were counted. The viral titers are expressed in PFU/mL.

### Preparation of BMDCs

The BMDCs were prepared as previously described (43). Bone marrow cells were isolated from mouse tibias and femurs. The cells were cultured for 7–9 days in a medium containing the mouse cytokine GM-CSF (20 ng/ml) to induce BMDCs. The positive rate was determined to be 85% by flow cytometry. The BMDCs collected were used for subsequent viral infection.

### SiRNA transfection

Bone marrow-derived dendritic cells (BMDCs) were transfected with siRNA at a final concentration of 10 μM, along with 2.5 μL of Lipofectamine™ RNAiMAX (Invitrogen, CA, USA), using Opti-MEM Reduced Serum Medium (Gibco) in a 12-well plate format. Following a 24-hour incubation period, MHV-A59 was introduced to the wells, which had been previously transfected with either target-specific siRNA or a negative control siRNA. All the siRNAs used were synthesized by JIMA Gene (China). The siRNA primers are listed in **Supplementary Table 9**.

### CRISPR validation

Single-guide RNAs (sgRNAs) were cloned into the CRISPR-Cas9 vector pLenti-CRISPR-V2 plasmid (98290, Addgene plasmid), sgRNA sequences are listed in **Supplementary Table 11**. Constructed plasmids were co-transfected into HEK-293T cells with packaging plasmids psPAX2 (12260, Addgene plasmid) and pMD2.G (12259, Addgene plasmid) at a ratio of 4:3:1. The culture medium was collected and filtered at 48h and 72h after transfection. The resulting supernatant containing lentiviral particles was used to infect the BMDCs seeded in 6 well plates, cells were infected twice to obtain higher transduction efficiency. After 48h infection, the medium supernatant was replaced with 5% FBS DMEM, the MHV-A59 was introduced to the wells, MOI=1. Cells were harvested for real-time quantitative PCR analysis, while supernatants were collected and centrifuged (500 ×g, 10 min) for plaque assays to quantify infectious viral particles at 24 hours post MHV infection.

### RNA isolation and quantitative RT-PCR

Total RNA was isolated from cells with TRIzol reagent in accordance with the manufacturer’s instructions. The mRNAs were reverse-transcribed into cDNA using the PrimeScript™ RT Reagent Kit (Takara). The cDNA was amplified using a fast two-step amplification program using SYBR Green Fast qPCR MasterMix (Cat No. 11201-11203*; Yeasen, China). GAPDH was used to normalize the input samples via the ΔΔCt method. The RT-PCR primers are listed in **Supplementary Table 10**.

### Quantitative transcriptomics and RNA-seq data processing

For the transcriptomic study, BMDCs were infected with MHV at the MOI=1, 1×10^6 cells were collected for each replicate, and total RNA was isolated, each replicate used 1μg total RNA as input for the transcriptomics. Eukaryotic mRNA was enriched with magnetic beads with Oligo (dT), and then, Fragmentation Buffer was added to break the obtained mRNA into short fragments. Using the post-fragmentation mRNA as a template, random primers were used to synthesize cDNA. After the ends of the obtained cDNA had been repaired, the sequencing primer was added. After amplifying the fragment by PCR, it was sequenced on the machine. cDNA libraries were generated and then sequenced on the Illumina Hiseq-2500 platform to generate 150bp PE (Paired-End) reads. Libraries were prepared using the Illumina TruSeq Stranded mRNA Library Prep Kit following the manufacturer’s protocol. Ligation adapters sequences: AGATCGGAAGAGCACACGTCTGAACTCCAGTCA, AGATCGGAAGAGCGTCGTGTAGGGAAAGAGTGT. The raw RNA-seq reads were trimmed using cutadapt (v1.13) to remove the adaptor sequences AGATCGGAAGAGCACACGTCTGAACTCCAG and AGATCGGAAGAGCGTCGTGTAGGGAAAGAG, and mapped to the Mouse Genome (mm10) using STAR (v2.5.3a) with GENCODE (vM18) gene annotations. The number of reads mapped to each gene was calculated using HTSeq (v0.11.2). Differential gene transcriptions were analyzed using DESeq2 (v1.18.1) with log2 (fold change) > 3.

### Protein isolation, digestion, and labeling with dimethylation reagents

BMDCs were infected with MHV at the MOI=1, 1×10^6 cells were collected for each replicate, each replicate used 30μg protein as input for the proteomics. The cell pellets were washed with ice-cold PBS and resuspended in 10 volumes of cell lysate (6 M urea/2 M thiourea containing protease inhibitor and phosphatase inhibitor). The cells were lysed using ultrasound and then centrifuged at 20,000 g for 20 min at 20 °C, and the supernatant was transferred to a new EP tube. The protein concentrations were determined by the Bradford assay. DTT was added to a final concentration of 10 mM, and the samples were incubated at 56 °C for 60 min before being cooled to room temperature. Iodoacetamide was added to a final concentration of 50 mM, and the mixture was incubated in the dark at 37°C for 45 min. Two hundred micrograms of protein were added to the Microcon 10 KD filter column, and 50 mM TEAB was added to a sample of 450 μl. This was centrifuged at 13,800 g and 25°C for 40 min. After that, the mixture was washed three times with 50 mM TEAB. The filter column was transferred to a new tube, and 200 μl of 50 mM TEAB and 4 μg of trypsin were added for digestion overnight at 37 °C. TFA was added to a final concentration of 0.5%. The digested peptides were collected by centrifugation at 13,800 g for 20 min. Two hundred microliters of 10% ACN was added to the filter column, which was washed with shaking for 15 min, and the solution was collected by centrifugation. The peptides obtained were dried with a Speed-Vac and dimethyl-labeled with CH_2_O (light labeled, the mock sample), CD_2_O (medium labeled, virus infection for 16 hours), and ^13^CD_2_O (heavy labeled, virus infection for 24 hours). The dimethyl-labeling experiment was performed by following a previously reported protocol. The light-labeled, medium-labeled, and heavy-labeled peptides were mixed at a ratio of 1:1:1 and separated by high-pH chromatography into 10 fractions.

### High-pH RPLC fractionation

Two hundred micrograms of peptides were fractionated using a Phenomenex Gemini^®^ 3 µm NX-C18 110 Å, 150 × 2 mm LC column on an Agilent 1200 Series high-pressure liquid chromatography (HPLC) system, operating at a flow rate of 0.3 mL/min with two buffer lines: Buffer A was 10 mM NH_4_HCO_3_; Buffer B consisted of 80% ACN and 10 mM NH_4_HCO_3_, pH 8. Samples were initially loaded onto the column at 0.3 mL/min for 10 min, after which the following fractionation gradient was commenced: 0% B to 50% B in 25 min, ramping to 100% B in 5 min, and the gradient was held at 100% B for 5 min before being ramped back to 0% B, where the column was then washed and equilibrated. Fractions were collected at 60 s intervals. Minutes 1–10 were pooled and used to represent 1 fraction. They were concatenated with other fractions as described in a previously reported protocol. With this method, we finally separated each sample into 10 fractions. All the samples were dried in a Speed-Vac and stored at −80°C until the LC-MS/MS analysis.

### Nano LC-MS/MS analysis and data processing

The samples obtained were analyzed in triplicate by Nano LC-MS/MS. Tryptic peptides were dissolved in 0.1% formic acid and loaded onto a trap column (Acclaim PepMapR 100, 100 μm × 2 cm, nano Viper C18, 5 μm, 100 ˚A) at a flow rate of 5 μL/min using an EASY-nLC 1000 system (Thermo Scientific, Odense, Denmark). Peptides were subsequently eluted from the trap column onto an analytical column (Acclaim PepMapR RSLC, 75 μm × 25 cm, nanoViper C18, 2 μm,100 ˚A) at a flow rate of 250 nl/min with a 60 min gradient: 3 to 8% Solvent B (A = 0.1% formic acid; B = 99% ACN, 0.1% formic acid) over 1 min, 8 to 22% Solvent B over 38 min, 22 to 30% Solvent B over 9 min, and 30 to 90% Solvent B over 2 min. Then, it was kept constant at 90% Solvent B for 10 min. Peptides eluted from the nanoLC were analyzed in a Q Exactive HF mass spectrometer system (Thermo Scientific, San Jose, CA, USA). The mass spectra were acquired in positive mode, and the data were acquired in DDA mode. The Orbitrap mass analyzer scan resolution was set to 60,000, and the full scan target was 3 × 10^6^ with a maximum fill time of 20 ms. The mass range was set to 300–1800 m/z. The 20 most intense ions were selected for MS/MS acquisition in the Orbitrap. The resolution of the MS/MS was set to 30,000. The target value for the fragment scans was set at 1 × 10^5^ with a maximum injection time of 45 ms. The isolation width was set to 1.6. The collision energy was set to 27, and the dynamic exclusion time was set to 30 s.

The MaxQuant software suite (61) (version 1.5.2.8) was used to identify proteins from a human database (Uniprot HUMAN, 2018_07, Entry number:16931) The 30 Raw files obtained by selecting the mass spectrum were uploaded to the MaxQuant software. The mass spectrometry data of three biological replicates were, respectively, labeled as Experiment 1, Experiment 2, and Experiment 3. For the database searches, the precursor mass tolerance was set to 20 ppm. The instruments selected, Orbitrap, PSM FDR, and Protein FDR, were set to 0.01. The other parameters were set to their default values. The quantitative method was set to 3 labels. Light labels were used for DimethLys0 and DimethNter0, Medium labels were used for DimethLys4 and DimethNter4, and Heavy labels were used for DimethLys8 and DimethNter8. The search included the fixed modification of carbamidomethyl (C) and variable modifications of oxidation (M) and acetyl (Protein N-term). A fully tryptic specific search was used. The maximum number of missed cleavages was set to 2.

### Bioinformatic analysis

Further analysis of the MaxQuant-processed data was performed using the Perseus software (version 1.6.1.1). The “proteingroups.txt” file produced by MaxQuant was loaded into Perseus for preprocessing. Then, bioinformatic analysis was performed using various types of bioinformatic software. The data correlation and heatmaps were analyzed using the Perseus software. We identified proteins that changed by more than 1.5 times in 0 or 24 hours compared to 0 hours (control) as being significantly changed. The lists of significantly changed proteins were uploaded to the PANTHER website (http://www.pantherdb.org/) for gene ontology analysis. The lists were also uploaded to the DAVID website (https://david.ncifcrf.gov) for functional clustering analysis, FDR ≤ 0.05, gene set size was set from 5 to 100. To determine the protein network involved in different clusters, a list containing the proteins from different clusters was uploaded to the STRING website (https://string-db.org). We put the top100 DEGs into analysis, the confidence score cutoff ≥ 0.7 (high confidence), pValue cutoff ≤ 0.05, interaction sources are from the database annotations. The output network was visualized using Cytoscape (version 3.4.0). We also used the KEGG database (https://www.kegg.jp) to analyze the data.

### Statistical analysis

Data were analyzed using the GraphPad Prism 5 software (GraphPad, La Jolla, CA, USA). *P < 0.05, **P < 0.01, and ***P < 0.001 denote significant differences.

## Data Availability

The raw RNA-seq data generated in this study have been deposited in the NCBI Sequence Read Archive (SRA) under BioProject accession number PRJNA1208741. The mass spectrometry proteomics data are available through the ProteomeXchange Consortium via the PRIDE partner repository under dataset identifier PXD061826.
