# Supplementary material for: Whole-cell response of coronavirus-infected BMDCs through proteomic and transcriptomic analyses
Source: Front Immunol. 2025 Jun 6;16:1513952. doi: 10.3389/fimmu.2025.1513952 (PMC12180443; doi:10.3389/fimmu.2025.1513952)

Supplementary Fig. 1. Flow cytometric analysis of isolated BMDC stimulated by GM-CSF.

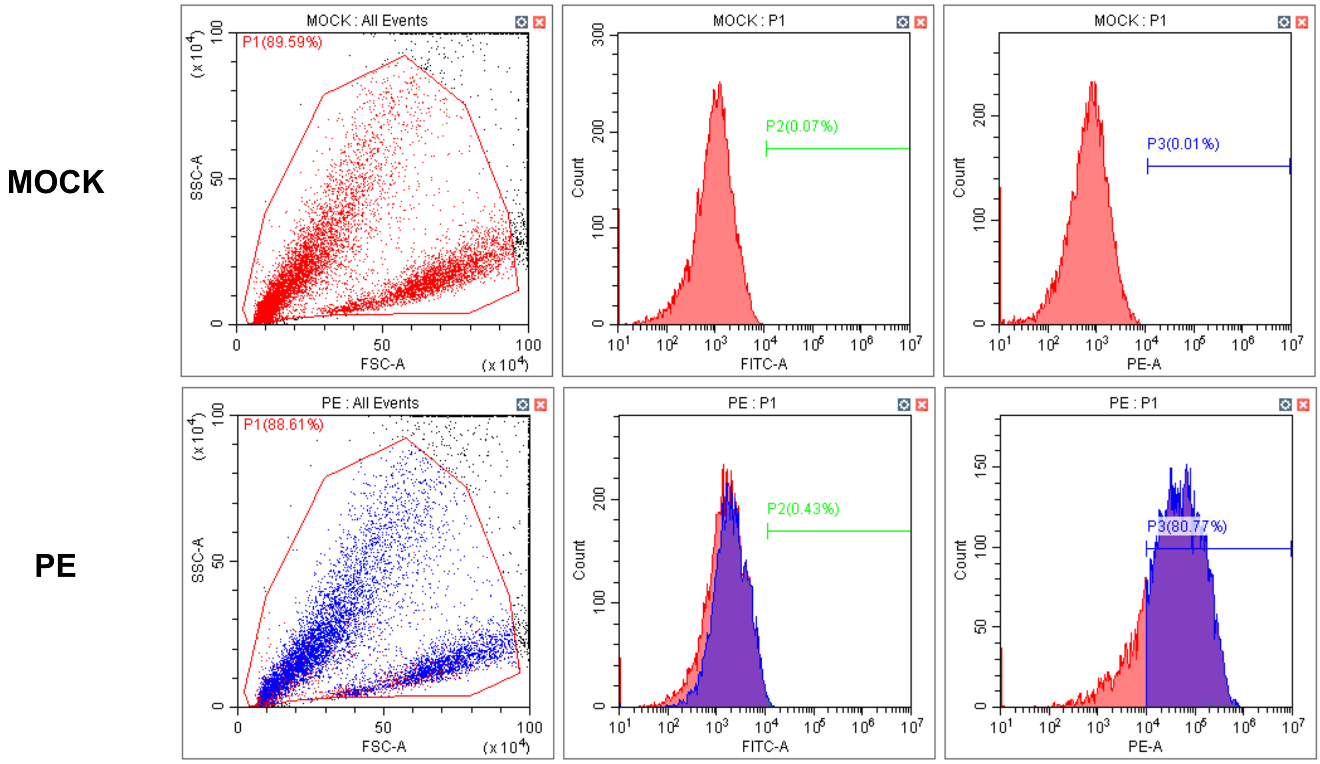

Supplementary Fig. 2. Quantitative proteomic and transcriptomic analyses of murine-hepatitis-virus-infected BMDCs.

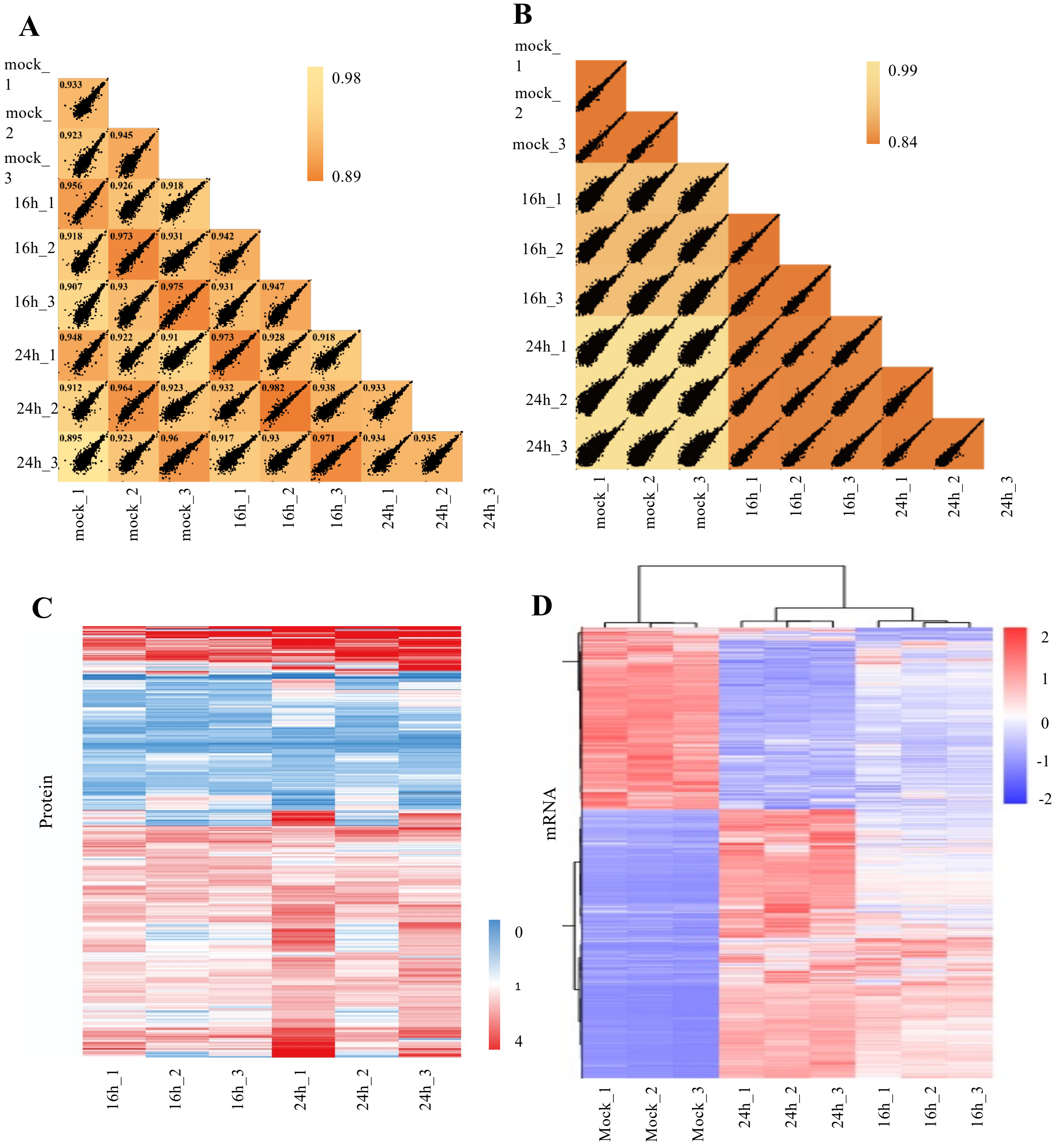

Supplementary Fig. 3. Volcano plot of differential gene expression in MHV-infected BMDCs.

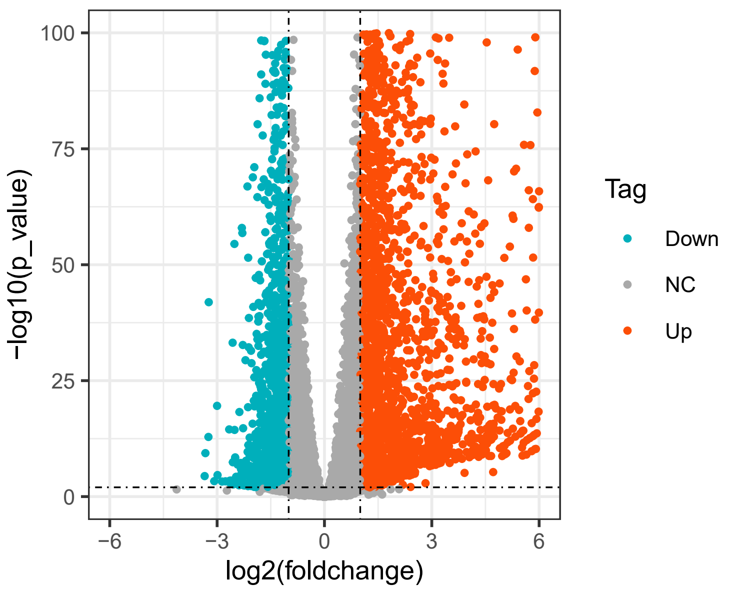

Supplementary Fig. 4. Validation of Differentially Expressed Genes by RT-qPCR.

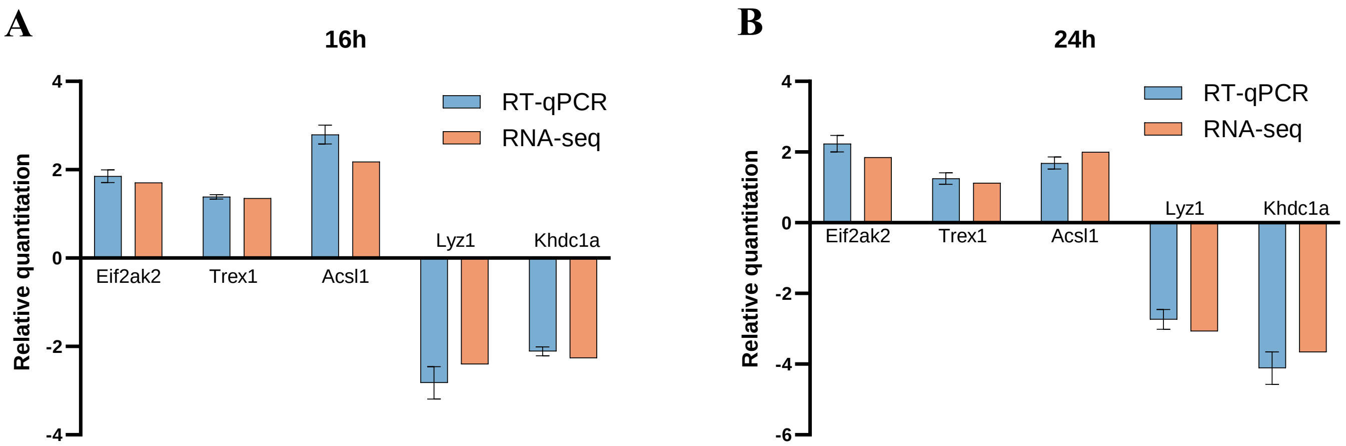

Supplementary Fig. 5. Proportion of co-upregulated and co-downregulated genes in the overlapping genes of transcriptome and proteome.

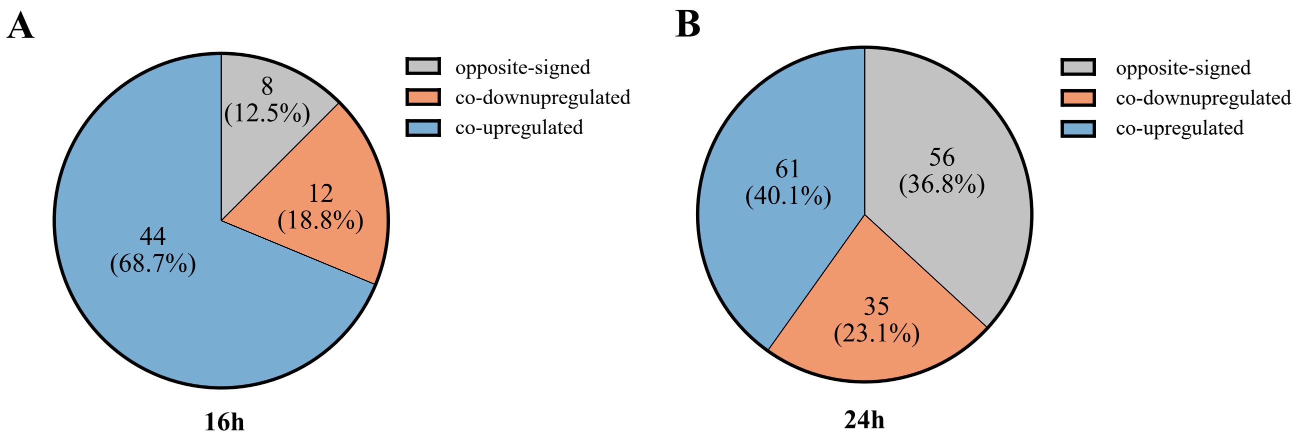

Supplementary Fig. 6. Functional analysis of regulated genes in the transcriptome of murine-hepatitis-virus-infected BMDCs.

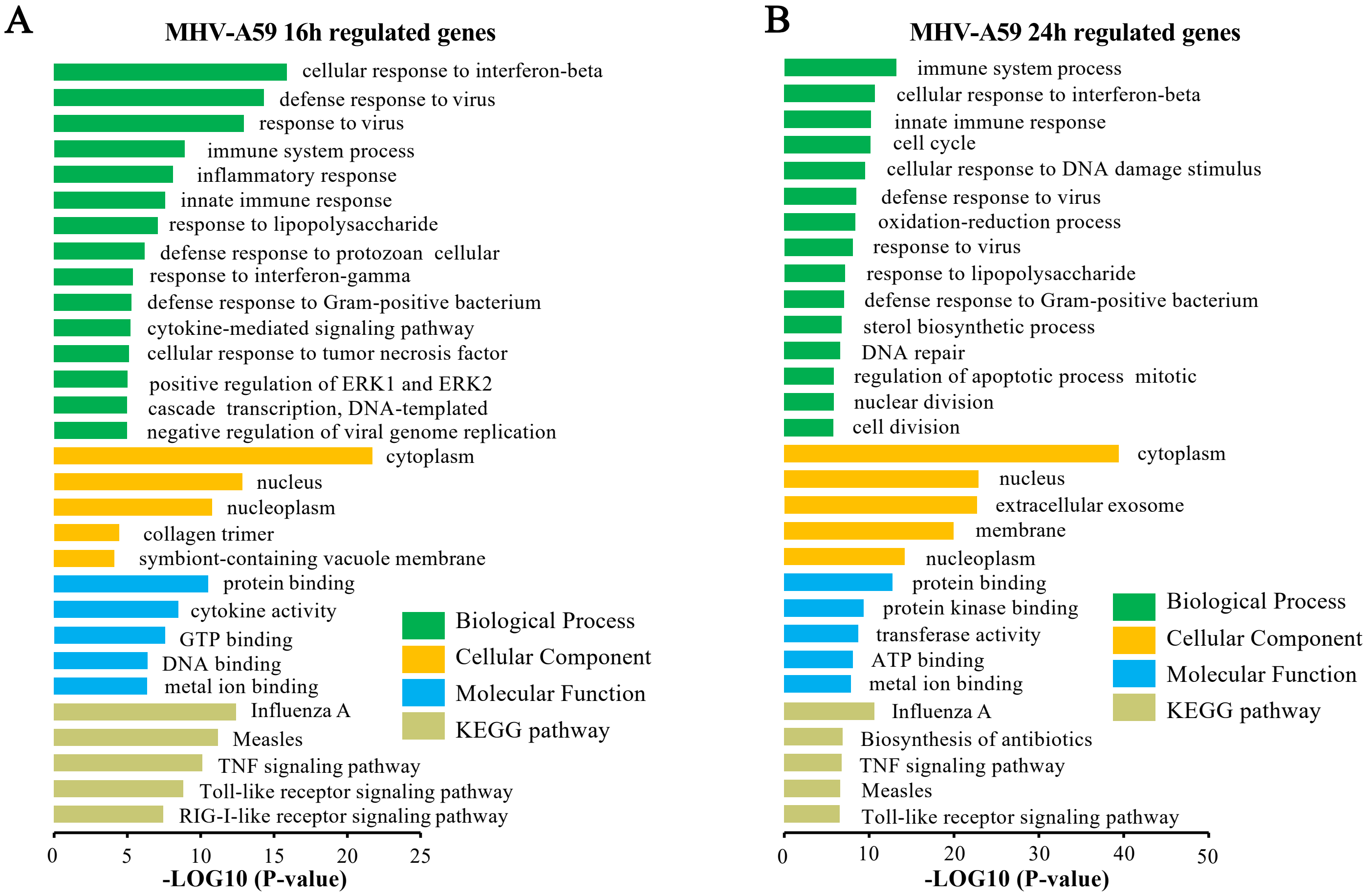

Supplementary Fig. 7. Comparative analysis of transcriptome data for SARS-CoV-2-infected Calu3 cells and DCs obtained from the database and our transcriptome data.

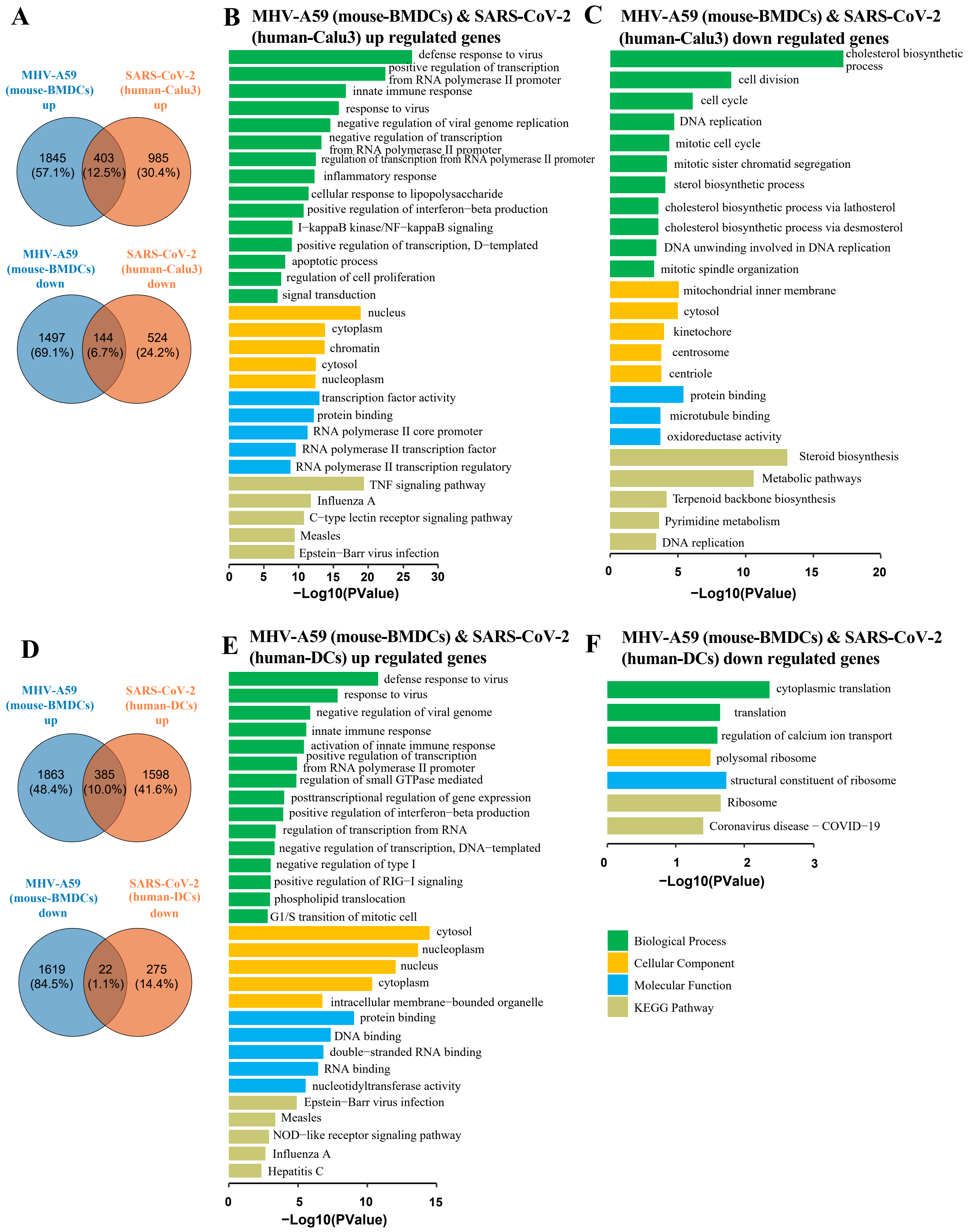

Supplementary Fig. 8. Analysis of co-variation factors among BMDCs, BMDMs and PMs after MHV infection.

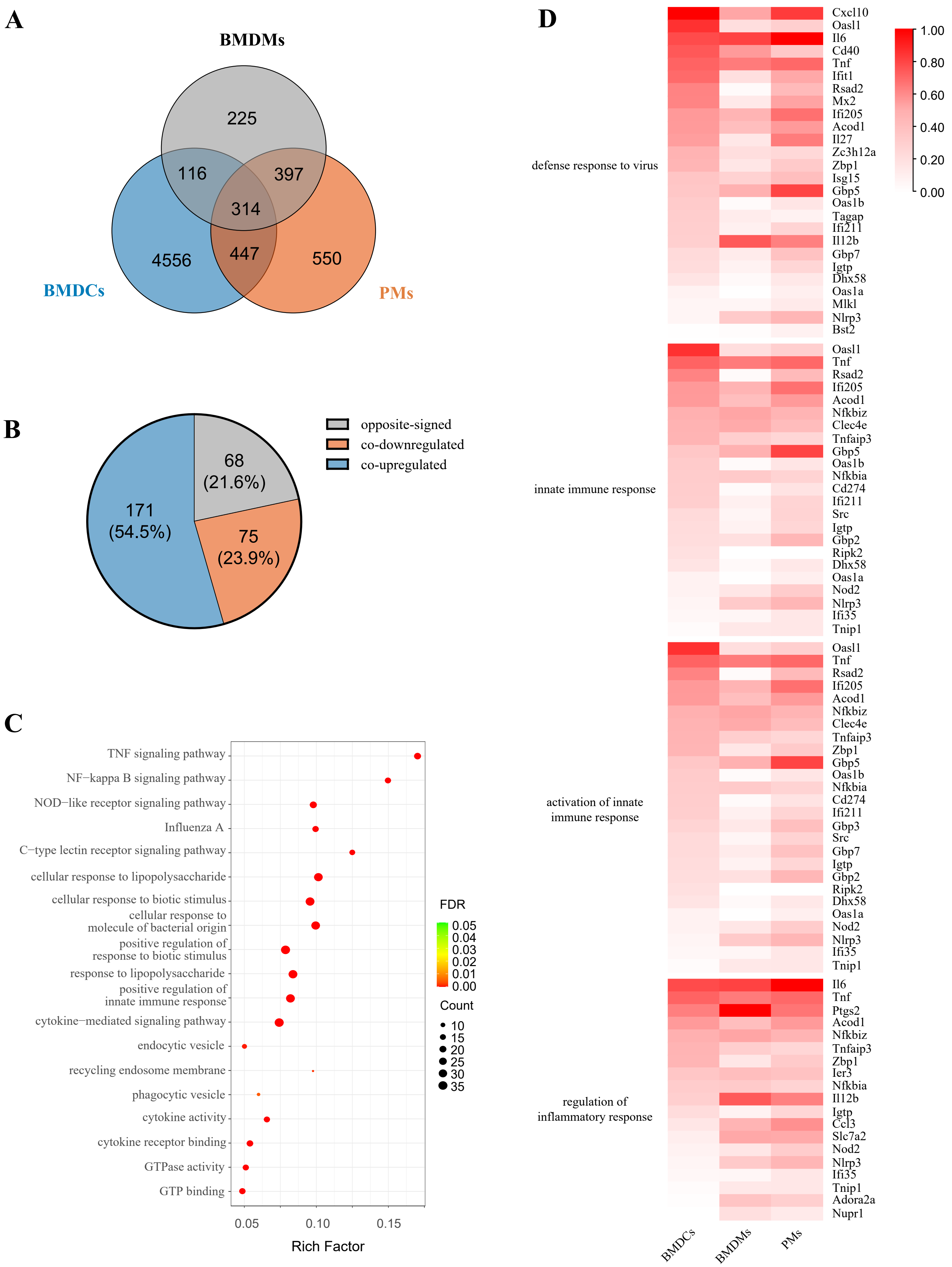

Supplementary Fig. 9. Verification of the antiviral function of selected genes from Fig. 3 and Fig. 6 via CRISPR-Cas9-mediated gene editing.

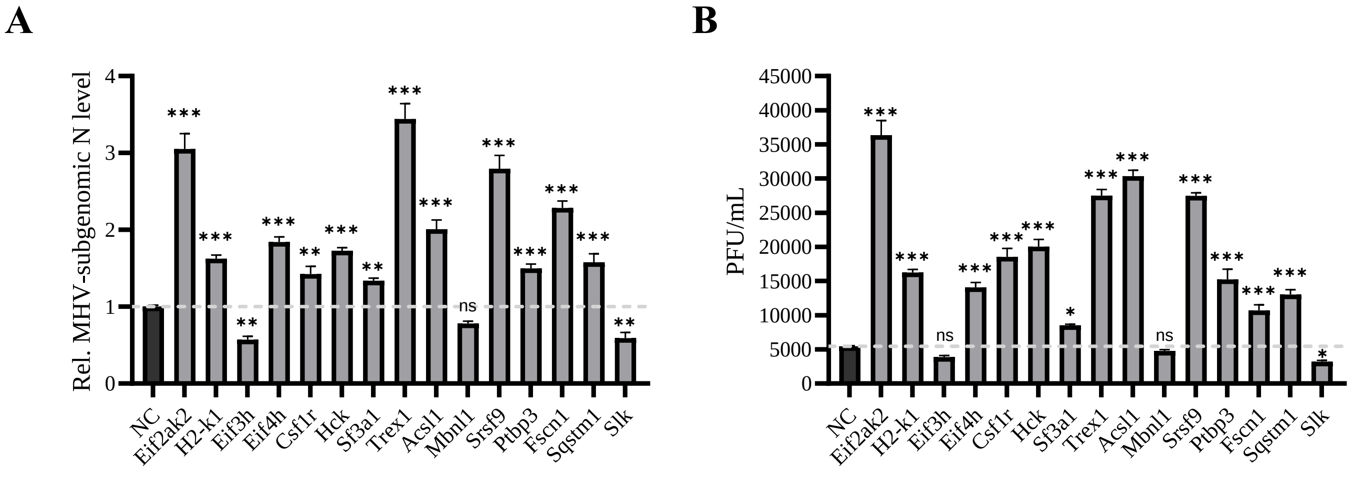

Supplement: Supplementary file 1 [file DataSheet1.pdf]
